# Improved Adhesion of Gold Thin Films Evaporated on Polymer Resin: Applications for Sensing Surfaces and MEMS

**DOI:** 10.3390/s130607021

**Published:** 2013-05-28

**Authors:** Behrang Moazzez, Stacey M. O'Brien, Erika F. Merschrod S.

**Affiliations:** Department of Chemistry, Memorial University, St. John's, NL A1B3X7, Canada; E-Mails: bm3184@mun.ca (B.M.); smob61@mun.ca (S.M.O.)

**Keywords:** thin films, adhesion, elastic modulus, roughness, biomedical cantilever sensors, microfluidic sensors, SU-8, metal, gold

## Abstract

We present and analyze a method to improve the morphology and mechanical properties of gold thin films for use in optical sensors or other settings where good adhesion of gold to a substrate is of importance and where controlled topography/roughness is key. To improve the adhesion of thermally evaporated gold thin films, we introduce a gold deposition step on SU-8 photoresist prior to UV exposure but after the pre-bake step of SU-8 processing. Shrinkage and distribution of residual stresses, which occur during cross-linking of the SU-8 polymer layer in the post-exposure baking step, are responsible for the higher adhesion of the top gold film to the post-deposition cured SU-8 sublayer. The SU-8 underlayer can also be used to tune the resulting gold film morphology. Our promoter-free protocol is easily integrated with existing sensor microfabrication processes.

## 1. Introduction

Gold is often used in microfabricated devices due to its high electrical conductivity and optical reflectivity, combined with a low Young's modulus of 79 GPa [1]. It has been used widely in chemical and biochemical sensors as a signal transducer based on each of the abovementioned unique properties or their combinations [2]. For example, a gold thin film serves well as a surface enhanced Raman scattering (SERS) substrate in miniaturized microfluidic lab on chip devices due to its inert character, required for continuous online monitoring under chemical stresses enforced by environment [3,4]. There are likewise applications in surface plasmon resonance (SPR) sensors [5].

One of the main issues in the applicability of gold thin films in microfabrication technology is their weak adhesion to inert and commonly used glass and silica substrates even using adhesion promoting processes [6–8]. Oxidative metals such as chromium, used as an intermediate layer, can enhance gold adhesion, but because chromium diffuses to the gold surface and oxidizes, the top gold layer morphology and electrical properties are affected dramatically [6,9]. Polymeric materials have gained popularity within these areas due to their flexible processing, high chemical resistance and reduced fabrication costs. Hence Ge *et al.* [10] and Nordström [1] have studied the adhesion of Cu and Au, respectively, to photosensitive epoxies including SU-8.

SU-8 is a photosensitive polymer in common use as a structural material [1,11], not only as a negative photoresist for subsequent fabrication steps. Nordström *et al.* fabricated cantilevers for biochemical detection systems with SU-8 [1]. In order to be able to coat the cantilevers with probe molecules, they deposited a thin layer of Au on top of the SU-8 surface. Sameoto *et al.* have also investigated methods to enhance gold adhesion to SU-8 to determine the best processing conditions to produce reliable electrical connections to SU-8 microelectromechanical systems (MEMS) [12].

Increasing gold adhesion to a glass substrate and/or SU-8 is also of interest for microfluidic based SERS substrates, especially if achieved in a more cost- and time-effective manner in comparison with recently patented methods [13] while preserving the same level of control over morphology and thermo-mechanical properties. More generally, adhesion of thin films is a vital consideration for sensor device performance. There are a few studies regarding gold deposition with physical vapor deposition methods on SU-8 such as reverse imprinting [14–16], and — to the best of our knowledge — no previous study has taken the approach we present here.

This paper presents a new method (post-Au-deposition SU-8 polymerization) to improve adhesion and tune the morphology of thermally-evaporated gold films. This protocol results in thin films with appropriate smoothness for many delicate applications in optics for gold mirrors and gratings, cantilever fabrications for atomic force microscopy [17], biosensors [11,18–20] and chemical sensors such as SERS-active substrates [3]. The method also offers the opportunity to control the fine (nanometer) scale morphology or roughness of the film, which has added advantages for SERS activity. This paper also illustrates both pragmatic and fundamental approaches to characterize adhesion quality of metal films, an essential consideration in sensor fabrication and design.

## 2. Experimental

### 2.1. Substrate Preparation

Glass slides (Cole-Parmer precleaned 25 × 75 × 1 mm Plain) were rinsed with 95.5% ethanol, 30% acetic acid and nanopure water (Barnstead, 18.2 MΩ·cm), dried with filtered, dry compressed air, and cut into 25 × 25 mm squares with a diamond scribe (SPI Supplies). After depositing 1 mL of SU-8 (Microchem Corp, SU-8 2010) photoresist onto a glass square, a two step spin-coating process (WS 400, Laurell Technologies) spread SU-8 evenly on the surface to give a final thickness of 20 *μ*m. Following acceleration of 100 rpm/s to a final spin speed of 500 rpm for 30 s, a second acceleration of 300 rpm/s led to the final spin speed of 1,000 rpm for 60 s.

Additional samples were prepared with final spin speeds of 4,000 rpm and 1,500 rpm, yielding film thicknesses of 10 *μ*m and 15 *μ*m, respectively. These samples were used to study the tunability of surface roughness.

A soft-bake step involved placing the coated glass slide on a hot plate (Corning PC-35) at 65 °C for one minute followed by 2 minutes on a hotplate (VWR 825 digital Aluminum top) at 95 °C, to evaporate the solvent. Exposure to UV light was performed on the stage of a Maskless Patterning System (Intelligent Micropatterning, LLC) for 2 min, immediately followed by a post-exposure bake following the same steps of soft bake for polymerization to occur.

The glass substrates of “pre-deposition cured” type followed the above steps in succession. The other type of substrate, the “post-deposition cured” type, involved a metal evaporation step after the soft-bake, before UV exposure.

### 2.2. Metal Deposition

Metal deposition was done using a conventional thermal evaporation chamber (built in-house) with a quartz crystal microbalance (QCM) thickness monitor (INFICON XTM/2 deposition monitor). Substrates, prepared as mentioned above, were placed on the sample holder disc and mounted 20 cm above the source at the same level as the QCM element. Gold foil (Johnson Matthey and Mallory gold) was placed in a tungsten boat (Kurt J. Lesker). Evaporation was conducted at pressures below 10^−3^ Pa. Substrates were removed after allowing the system to cool down to ambient temperature to avoid carbon contamination usually caused by introduction of air to a hot chamber. A 10 nm layer of gold was evaporated on the polymer coated glass slides as reported by the QCM thickness measurement unit. Film thickness was measured independently by atomic force microscopy (Asylum Research MFP-3D system with MikroMasch CSC37/Cr-Au tip) to be 12 nm.

### 2.3. Mechanical Properties Measurements

Initial screening of adhesion quality involved two simple tests: manual scratching with a tweezer (SPI Swiss Wafer Tweezers 4WF), and attachment/removal of adhesive tape (3M Scotch Magic Tape 810, 3/4 in wide). Then a progressive load scratch test (PLST) was performed with an MFP-3D (Asylum Research) atomic force microscope in contact mode using a silicon tip (NSC35/A1BS, nominal spring constant 6.5–27.5 N/m, MikroMasch). In order to grade the adhesion quality in a quantified manner, increasing forces were applied while the AFM tip was scanning the sample in contact mode until the tip deflection signal showed a discontinuity, also seen in the height and lateral force signals and as a scratch under the optical view. To apply a variable force, the deflection set point was increased as the tip scanned a 90 *μ*m × 90 *μ*m area. The cantilever deflection set point value is measured as a voltage difference from a split photodiode that detects a laser beam reflected off the back of the cantilever. The force required to scratch is directly proportional to the deflection of the cantilever (measured as a voltage between 0 and 10 V) at the initiation point of the scratch.

To further assess the mechanical response of the gold thin films on the microscale, a combination of destructive nanoindentation and contact mode imaging was performed using the same type of probes as above (NSC35/A1BS, MikroMasch). Pre-indentation contact mode images of a 40 *μ*m × 40 *μ*m area with scan rate of 1 Hz were followed by destructive indentation (to depths of at least 100 nm, thus past the gold layer and into the SU-8 underlayer) at different locations within that area. Post-indentation contact mode images were obtained of the indented regions. In addition to morphological information, this further identified samples with poor adhesion and hence vulnerable to disruption by the cantilever during scanning.

Embedded functions within the MFP3D software (Asylum Research) were used to measure stiffness and elastic modulus at regular intervals across a sample. Force maps (50 points over a 20 *μ*m × 20 *μ*m area for the post-deposition cured sample and 48 points over a 20 *μ*m × 20 *μ*m area for the pre-deposition cured sample; indentation rate of 1.39 *μ*m/s) were acquired to extract elasticity data. The force set point was selected for tip indentation depths of around 2 nm (one fifth of the thickness of gold film), to acquire data that reflect the properties of top gold film without also directly probing the underlying solid support (glass slide), and to avoid the plastic deformation described above. Sample force curves are provided in the Supplementary Material (Figures S1 and S2).

Before indenting the sample, force curves were collected on a bare mica substrate to calibrate the deflection sensitivity of the instrument. For proper selection of cantilever stiffness with regard to sample stiffness, different levers with different spring constants were tried to choose the lever that produces appropriate force curves. The lever of choice has a spring constant of 20 N/m, as measured using the thermal noise method [21]. All force data used here were obtained with the same tip.

Elastic moduli were calculated based on the Hertzian model [22] by extracting force and indentation depths from the retraction segment of each of the fitted force curves [23], assuming a gold Poisson's ratio of 0.42 [24] Stiffness was calculated as the slope of the retraction curve, the applied force required to achieve a given indentation.

### 2.4. Topography Measurements

AFM topography images from which we obtained our roughness data were acquired under contact mode (CSC37/AuCr tip, MikroMasch) with a scanning area of 10 *μ*m × 10 *μ*m and scan rate of 1 Hz. Roughness was calculated as the root mean square height value for each image:

(1) $$\text{Roughness}=\sqrt{\frac{1}{N}\sum_{i}Y_{i}^{2}}$$

N is the number of points (pixels in the image) and Y is the height at each pixel.

## 3. Results

### 3.1. Scratch Tests

Tweezer scratching and tape attachment/removal tests indicate that the post-deposition cured sample is more robust than the pre-deposition cured one. The following PLST results verify and quantify this difference in film adhesion quality

**Figure 1.**
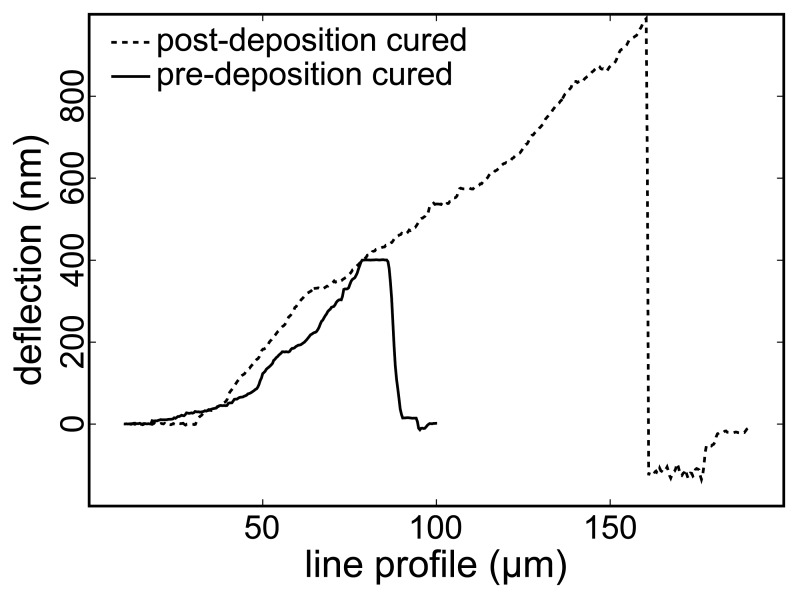
Line profile (perpendicular to the fast scan direction) across the deflection retrace image that is obtained while the set point voltage is manually increased. The post-deposition cured sample requires a much higher applied force (and hence tip deflection) before film disruption (scratching) occurs.

Figure 1 is a line profile, perpendicular to the fast scanning direction, from the deflection retrace data channel. This plot clearly illustrates the initiation point of the scratch, when the deflection signal drops after reaching a maximum. The deflection increases as the set point value is increased until the point when the tip penetrates the gold film and the scratch begins, where the deflection drops immediately from its maximum and stays at the same dropped value as the tip pulls away the gold layer.

The higher the voltage for the set point value just before scratch starts, the higher would be the deflection of the cantilever, and both are proportional to a larger force being applied by the cantilever tip against the film surface to initiate the scratch. The set point voltage at the initiation of the scratch for the post-deposition cured sample was at least 1 *μ*m or 10 V (the maximum measurable deflection) and for the pre-deposition cured sample falls in the range of 320–420 nm or 3.2–4.2 V. This indicates that at least 2.4 times higher force is required to initiate a scratch on the post-deposition cured sample. It is worth mentioning that for a gold thin film deposited on bare glass, scratching starts at a set point of 0.5 V or 50 nm deflection.

### 3.2. Indenting-Imaging Tests

**Figure 2.**
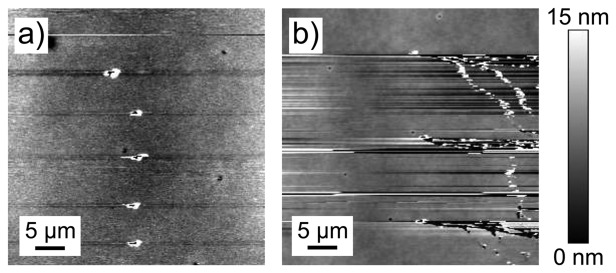
Contact mode images after indentation tests for a post-deposition cured sample (**a**) and a pre-deposition cured sample (**b**). Image (b) shows streaking: gold prepared with pre-deposition curing is so fragile that when the tip reaches the indented area film it starts to scratch. In contrast, indentations could be clearly imaged for post-deposition cured sample (a).

In the contact mode image for a post-deposition cured sample (Figure 2(a)) the tiny black holes with white color shells are indented locations where the white shells are debris due to disruption of the gold film. The image shows clear signs of deformation during indentation, reinforcing the need to extract elastic data from the retraction and not the extension curve.

The indentation marks are not clear in the pre-deposition cured sample (Figure 2(b)). Pre-deposition cured films demonstrate a delicate structure, being less scratch-resistant compared with post-deposition cured films. For post-indentation imaging, when the tip reached the indented region of the pre-deposition cured film (Figure 2(b)), it started to scratch the gold surface while imaging.

### 3.3. Force Curves

Average indentation depths, elastic moduli, and stiffness are collected in Table 1. A shallower indentation value of 2.3 ± 0.4 nm was observed for post-deposition cured thin film samples compared with 2.8 ± 0.8 nm for the pre-deposition cured samples, with a correspondingly higher stiffness. The difference in average stiffness and indentation depth between these two sets of data is statistically significant with 95% confidence (P_stiff_ = 2 × 10^−4^; P_depth_ = 6 × 10^−4^). Here and elsewhere, assessment of statistical significance between populations uses a two-tailed t-test assuming unequal variance in the two data sets.

**Figure 3.**
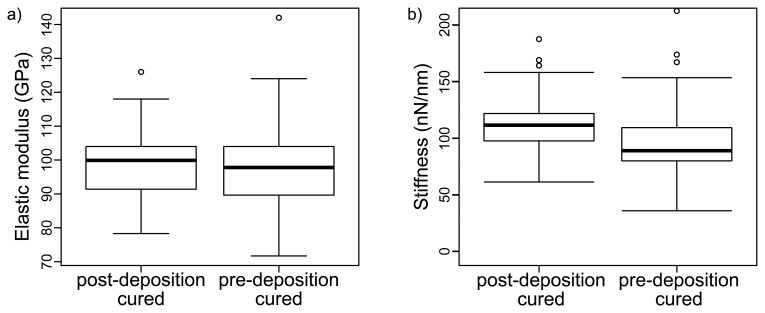
Box plots for elastic modulus (**a**) and stiffness (**b**) values calculated for each sample. The average elastic moduli for the two sample types are not significantly different, but the stiffness for the post-deposition cured sample is significantly higher. The circles are outliers.

Figure 3(a) shows the distribution of calculated elastic properties for each sample with box plots. The mean elastic modulus is slightly larger for the post-deposition cured gold film sample, but a t-test analysis rejects any significant difference, rather providing a significant (19%) probability of similarity Gold films prepared with the pre-deposition recipe give more centered values but a wider range for elastic modulus, while clearly for post-deposition cured samples the values are distributed in slightly narrower range although skewed. The stiffness values shown in Figure 3(b) do differ significantly, with the post-deposition cured sample demonstrating higher stiffness and a narrower distribution of stiffness. The force curves were shallow enough that they showed no signatures of plastic deformation.

Because we are measuring thin films, there is a chance that the substrate contributes significantly to the resultant nanoindentation measurement. Analysis of curves with indentation depths less than 2 nm (to avoid excess contribution from the solid support) yields elastic moduli of 93 ± 10 GPa for the post-deposition-cured sample and 101 ± 23 GPa for the pre-deposition cured sample, with distributions with significant overlap with the whole data set. Furthermore, the force curve shapes indicate that the measurements were not affected by the underlying substrate: the curvature of the extension and retraction curves was accounted for entirely by probe shape and did not show evidence of coupling with the harder substrate. The solid support cannot be excluded entirely given the thin and soft nature of the film being measured [25], but this small contribution will be found across all samples and therefore the trends we measure are still interpretable.

### 3.4. AFM Topography Images

**Figure 4.**
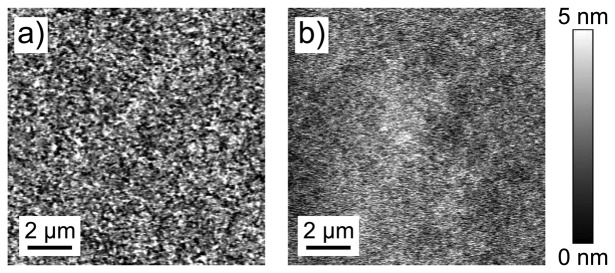
AFM images for gold films prepared by the (**a**) post-deposition cured recipe and (**b**) pre-deposition cured recipe on 20 *μ*m thick SU-8 layers. The morphologies and hence roughness values are different.

Height images for both pre-deposition cured and post-deposition cured samples (Figure 4) show generally smooth films (roughness values on the order of 1 nm). Sample roughness can introduce distortion in the force curve due to torsional momentum resulting in a twist of the tip [26], but our samples with roughness of less than 3 nm on a 1 *μ*m × 1 *μ*m scale will not show this effect.

A gold film on bare glass has a roughness of 1.42 nm (as defined in Equation (1)). The pre-deposition-cured films show a much smoother gold layer, while the post-deposition-cured films are rougher. Table 2 presents these data for gold films on a 20 *μ*m-thick SU-8 underlayer, the sample type discussed up to this point. Data from additional samples with varying thickness of SU-8 underlayer (15 *μ*m and 10 *μ*m) show broader tunability of the gold layer morphology for the post-deposition-cured samples.

## 4. Discussion

Thin-film coatings may be non-durable under thermal fluctuations where materials with different thermal expansion coefficients are joined together in a packed sensor. Problems with mechanical stability can be exacerbated when one layer is deposited at elevated temperature as in the case of gold thermal evaporation, where the different thermal expansion coefficients of metal, underlayer and substrate can lead to substantial residual stress upon cooling [27]. Subsequent mechanical impacts, such as the scratch, tape and nanoindentation tests, can result in different types of film disruption depending on the balance between the elasticity of the film and the strength of interactions between the film and the underlayer.

Our morphological investigations indicate a stronger coupling between the underlayer and the gold film for the post-deposition cured film. The post-deposition cured sample shows larger scale (∼1 *μ*m) height fluctuations than the pre-deposition cured sample, which is smoother with feature sizes below 300 nm. One would expect higher yield stress for materials with smaller grain size [28], with all other things being equal, but the morphological features in the gold film are not individual grains in a polycrystalline film. In fact, the longer-scale corrugations and overall film morphology are unique to the post-deposition-cured film. This suggests that the polymer layer can convey morphology and contours to the top metal layer when cured after gold deposition.

By varying the thickness of the SU-8 underlayer, the roughness can be fine-tuned as well, as shown in Table 2. The data for both pre- and post-deposition cured methods demonstrate that the gold film morphology is independent of the glass substrate, with resulting roughness controlled by the SU-8 layer. The relation between SU-8 thickness and roughness is not simple, however. The roughness of pre-deposition-cured samples is quite similar regardless of SU-8 thickness, while there is much more variation for the post-deposition-cured samples. While the resulting gold film morphology likely reflects the morphology of the underlying SU-8, different morphologies emerge when the polymer curing takes place in the presence of the gold film. This coupling between the gold and polymer layers also plays a role in the resultant mechanical properties.

The elastic modulus values for both samples are around the 100 Gpa value which Moody *et al.* [9]. The pre- and post-deposition cured films do not differ significantly in elastic modulus. The large and overlapping distributions of elasticity values are consistent with the large scatter in mechanical properties measured by others for thin films at small indentation depths [29]. The shallower average indentation value required to reach the force set point for the post-deposition cured sample does indicate greater stiffness, which is in fact what we measure (see Table 1). The difference in spread between stiffness data and elastic modulus data (which is derived from stiffness) likely arises from differences between the idealized tip shape used to calculate all elastic moduli data and the real (and possibly changing) tip shape.

The plastic deformation seen after destructive nanoindentation for the post-deposition cured film in Figure 2(a) is in sharp contrast to the cracking and delamination of the pre-deposition cured film shown in Figure 2(b). The PLST experiments also demonstrate that the post-deposition cured film is significantly stronger (requires a higher load to rupture). AFM scratch tests (PLST) reveal better adhesion for gold films on post-deposition cured SU-8 polymer, and simple tweezer scratch and tape tests confirm this.

Interface structure and composition are two of the most important factors controlling the performance and reliability of thin film devices [30]. The data point toward a model in which, during post-deposition cross-linking, the SU-8 chains cross-link around the bottom layer of large and heavy gold atoms. This cross-linking and the subsequent shrinkage of polymerized SU-8 due to reduction in free volume trap the bottom layer of gold atoms, resulting in gold-polymer composite structure with overall good adhesion of the gold layer to the cured SU-8 sublayer and the observed robustness of the post-deposition cured film. The gold atoms also impact the post-deposition cross-linking, imposing a spatial disturbance for chains to cross-link completely just beneath the adjacent gold layer. Hence lightly cross-linked SU-8 chains in the vicinity of the gold layer lead to the increased plasticity observed with the destructive nanoindentation (Figure 2(a)).

For the post-deposition cured sample, stress introduced during cooling after evaporation can be reduced during this cross-linking stage. The additional stress from macroscopic shrinkage due to cross-linking is relieved through film corrugation: as the film shrinks laterally it can expand normal to the substrate. This creates the uneven film surface observed in the AFM image in Figure 4(a).

In contrast, the pre-deposition cured film is smoother and less robust. The coupling between the gold layer and the SU-8 underlayer is much weaker, and it cannot access the stress-reducing mechanisms available in the post-deposition curing process.

## 5. Conclusions

Post-gold-deposition curing of a polymer underlayer is a reliable protocol to have a robust gold thin film satisfying the mechanical requirements for sensors. This protocol can also address issues of subsequent processing/packaging required to develop biomedical cantilever sensors or robust gold substrates for SERS or SPR sensors, where the graded gold–polymer interface can better withstand the issues arising from the different thermal expansion coefficients of the constituents.

The data show that the post-deposition cured samples are more resistant to applied mechanical stresses. The smaller spread in data seen for indentation depths for the post-deposition cured sample is repeated for stiffness and elastic modulus measurements, further emphasizing the more homogeneous and reliable behavior for the post-deposition cured sample.

Finally, the film topography that is translated from the SU-8 sublayer could be of interest for creating a controlled roughness for the “hot spots” used in surface enhanced Raman sensing.

## Supplementary Material



## Figures and Tables

**Table 1.** Average indentation depths, elastic moduli and stiffness for each sample. Statistical information about these averages is provided in the text and in Figure 3.

**Sample**	**Indentation Depth** **(nm)**	**Elastic Modulus** **(GPa)**	**Stiffness** **(nN/nm)**
Pre-deposition-cured	2.8	98	98
Post-deposition-cured	2.3	100	113

**Table 2.** Roughness values for 12 nm thick gold films on pre- and post-deposition cured samples with different thickness of SU-8 underlayer. For comparison, the roughness measured for gold film without an SU-8 underlayer was 1.42 nm.

**Sample**	**Roughness in nm**		

	**20** *μ*m **SU-8**	**15** *μ*m **SU-8**	**10** *μ*m **SU-8**
Pre-deposition-cured	1.02	0.90	0.98
Post-deposition-cured	1.36	2.01	1.52

## References

[1] Nordstrom M., Johansson A., Sánchez Nogueron E., Calleja M., Boisen A. (2005). Investigation of the bond strength between the photo-sensitive polymer SU-8 and gold. Microelectron. Eng..

[2] Wilson D.M., Hoyt S., Janata J., Booksh K., Obando L. (2001). Chemical sensors for portable, handheld field instruments. IEEE Sens. J..

[3] Halvorson R.A., Vikesland P.J. (2010). Surface-Enhanced Raman Spectroscopy (SERS) for environmental analyses. Environ. Sci. Technol..

[4] Haynes C.L., Yonzon C., Zhang X., van Duyne R. (2005). Surface-enhanced Raman sensors: Early history and the development of sensors for quantitative biowarfare agent and glucose detection. J. Raman Spectrosc..

[5] Taguchi Y., Takano E., Takeuchi T. (2012). SPR sensing of bisphenol a using molecularly imprinted nanoparticles immobilized on slab optical waveguide with consecutive parallel Au and Ag deposition bands coexistent with bisphenol a-immobilized Au nanoparticles. Langmuir.

[6] George M.A., Glaunsinger W.S., Thundat T., Lindsay S.M. (1990). Electrical, spectroscopic, and morphological investigation of chromium diffusion through gold films. Thin Solid Films.

[7] Audino R., Destefanis G., Gorgellino F., Pollino E., Tamagno S. (1976). Interface behavior evaluation in gold/chromium, gold/titanium and gold/palladium/titanium thin films by means of resistivity and stylus measurements. Thin Solid Films.

[8] Kang K.D., Burgess R.R., Coleman M.G., Keil J.G. (1969). Chromium-silver-gold metallization system. IEEE Trans. Electron. Devices.

[9] Moody N.R., Adams D.P., Medlin D., Headley T., Yang N., Volinsky A. (2003). Effects of diffusion on interfacial fracture of gold-chromium hybrid microcircuit films. Int. J. Fract..

[10] Ge J., Kivilahti J.K. (2002). Effects of surface treatments on the adhesion of Cu and Cr/Cu metallizations to a multifunctional photoresist. J. Appl. Phys..

[11] Johansson A., Blagoi G., Boisen A. (2006). Polymeric cantilever-based biosensors with integrated readout. Appl. Phys. Lett..

[12] Sameoto D., Lee S.W., Parameswaran M. (2008). Wirebonding characterization and optimization on thick film SU-8 MEMS structures and actuators. J. Micromech. Microeng..

[13] Allara D.L., Dwight D.W. (2008). Surface enhanced raman spectroscopy (SERS) substrates exhibiting uniform high enhancement and stability.

[14] Cardozo B.L., Pang S.W. (2008). Patterning of polyfluorene based polymer light emitting diodes by reversal imprint lithography. J. Vac. Sci. Technol. B.

[15] Chen H.L., Chuang S.Y., Lee W.H., Kuo S.S., Su W.F., Ku S.L., Chou Y.F. (2009). Extraordinary transmittance in three-dimensional crater, pyramid, and hole-array structures prepared through reversal imprinting of metal films. Opt. Express.

[16] Peng C., Cardozo B.L., Pang S.W. (2008). Three-dimensional metal patterning over nanostructures by reversal imprint. J. Vac. Sci. Technol. B.

[17] Schneider A., Ibbotson R.H., Dunn R.J., Huq E. (2011). Arrays of SU-8 microcantilevers with integrated piezoresistive sensors for parallel AFM applications. Microelectron. Eng..

[18] Godin M., Tabard-Cossa V., Miyahara Y., Monga T., Williams P.J., Beaulieu L.Y., Lennox R.B., Grutter P. (2010). Cantilever-based sensing: The origin of surface stress and optimization strategies. Nanotechnology.

[19] Mertens J., Calleja M., Ramos D., Taryn A., Tamayo J. (2007). Role of the gold film nanostructure on the nanomechanical response of microcantilever sensors. J. Appl. Phys..

[20] Calleja M., Tamayo J., Nordström M., Boisen A. (2006). Low-Noise polymeric nanomechanical biosensors. Appl. Phys. Lett..

[21] Hutter J.L., Bechhoefer J. (1993). Calibration of atomic force microscope tips. Rev. Sci. Instrum..

[22] Hay J.L., Wolff P.J. (2001). Small correction required when applying the Hertzian contact model to instrumented indentation data. J. Mater. Res..

[23] Kumar R.M., Merschrod S E.F., Poduska K.M. (2009). Correlating mechanical properties with aggregation processes in electrochemically fabricated collagen membranes. Biomacromolecules.

[24] Kipp D.O. (2010). Metal Material Data Sheets.

[25] Guo S., Akhremitchev B.B. (2006). Packing density and structural heterogeneity of insulin amyloid fibrils measured by AFM nanoindentation. Biomacromolecules.

[26] Pratt J.R., Smith D.T., Newell D.B., Kramar J.A., Whitenton E. (2004). Progress toward Système International d'Unités traceable force metrology for nanomechanics. J. Mater. Res..

[27] Audoly B. (2000). Mode-dependent toughness and the delamination of compressed thin films. J. Mech. Phys. Solids..

[28] Volinsky A.A., Moody N.R., Gerberich W.W. (2004). Nanoindentation of Au and Pt/Cu thin films at elevated temperatures. J. Mater. Res..

[29] Du K., Pang X., Chen C., Volinsky A.A. (2008). Mechanical Properties of Evaporated Gold Films. Hard Substrate Effect Correction. Mater. Res. Soc. Symp. Proc..

[30] Moody N.R., Adams D.P., Volinsky A.A., Kriese M.D., Gerberich W.W. (2000). Annealing effects on interfacial fracture of gold-chromium films in hybrid microcircuits. Mater. Res. Soc. Symp. Proc..

