# Supplementary material for: Improved Adhesion of Gold Thin Films Evaporated on Polymer Resin: Applications for Sensing Surfaces and MEMS
Source: Sensors (Basel). 2013 May 28;13(6):7021–32. doi: 10.3390/s130607021 (PMC3715244; doi:10.3390/s130607021)
Supplement: Supplementary file 1 [file sensors-13-07021-s001.pdf]

OPEN ACCESS
sensors
ISSN 1424-8220
www.mdpi.com/journal/sensors

Supplementary Information

Improved Adhesion of Gold Thin Films Evaporated on Polymer Resin: Applications for Sensing Surfaces and MEMS. *Sensors* 2013, *13*, 7021-7032

Behrang Moazzez, Stacey M. O'Brien and Erika F. Merschrod S. *

Department of Chemistry, Memorial University, St. John's, NL A1B3X7, Canada

E-Mails: bm3184@mun.ca(B.M.); smob61@mun.ca(S.M.O.)

* Author to whom correspondence should be addressed; E-Mail: erika@mun.ca;
Tel.: +1-709-864-8765; Fax: +1-709-864-3702.

Figure S1. A typical force curve from the post-deposition-cured sample.

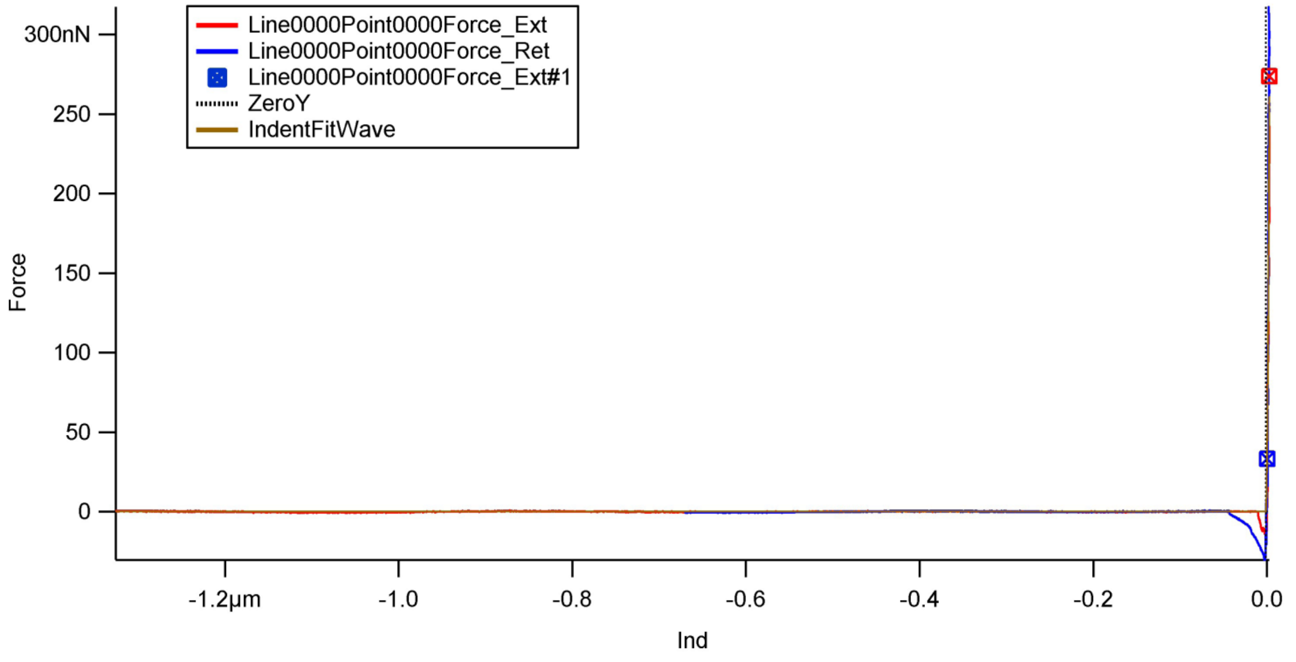

Figure S2. Magni_ed view of the same force curve from the post-deposition sample, showing the region fitted to calculate adhesion and elastic modulus.

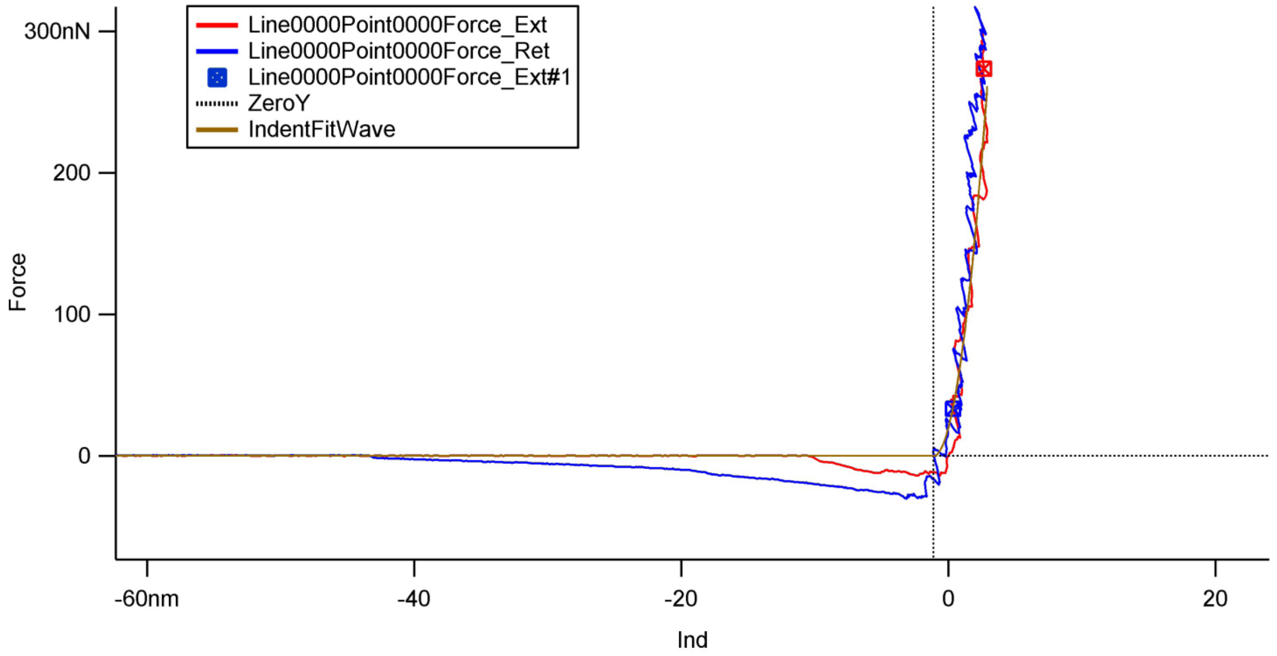

© 2013 by the authors; licensee MDPI, Basel, Switzerland. This article is an open access article distributed under the terms and conditions of the Creative Commons Attribution license (http://creativecommons.org/licenses/by/3.0/).
